# Whole body vibration for older persons: an open randomized, multicentre, parallel, clinical trial

**DOI:** 10.1186/1471-2318-11-89

**Published:** 2011-12-22

**Authors:** Mercè Sitjà-Rabert, Mª José Martínez-Zapata, Azahara Fort-Vanmeerhaeghe, Ferran Rey-Abella, Daniel Romero-Rodríguez, Xavier Bonfill

**Affiliations:** 1 Physiotherapy Research Group (GReFis), Blanquerna School of Health Science (Universitat Ramon Llull), Barcelona, Spain; 2 Iberoamerican Cochrane Centre. Institute of Biomedical Research (IIB Sant Pau); Universitat Autònoma de Barcelona; CIBER Epidemiología y Salud Pública, CIBERESP, Barcelona, Spain; 3 EUSES Health and Sport Sciences School, Universitat de Girona, Girona, Spain; 4 Public Health and Clinical Epidemiology Service. Hospital de la Santa Creu i Sant Pau, Barcelona, Spain

## Abstract

**Background:**

Institutionalized older persons have a poor functional capacity. Including physical exercise in their routine activities decreases their frailty and improves their quality of life. Whole-body vibration (WBV) training is a type of exercise that seems beneficial in frail older persons to improve their functional mobility, but the evidence is inconclusive. This trial will compare the results of exercise with WBV and exercise without WBV in improving body balance, muscle performance and fall prevention in institutionalized older persons.

**Methods/Design:**

An open, multicentre and parallel randomized clinical trial with blinded assessment. 160 nursing home residents aged over 65 years and of both sexes will be identified to participate in the study. Participants will be centrally randomised and allocated to interventions (vibration or exercise group) by telephone. The vibration group will perform static/dynamic exercises (balance and resistance training) on a vibratory platform (Frequency: 30-35 Hz; Amplitude: 2-4 mm) over a six-week training period (3 sessions/week). The exercise group will perform the same exercise protocol but without a vibration stimuli platform. The primary outcome measure is the static/dynamic body balance. Secondary outcomes are muscle strength and, number of new falls. Follow-up measurements will be collected at 6 weeks and at 6 months after randomization. Efficacy will be analysed on an intention-to-treat (ITT) basis and 'per protocol'. The effects of the intervention will be evaluated using the "t" test, Mann-Witney test, or Chi-square test, depending on the type of outcome. The final analysis will be performed 6 weeks and 6 months after randomization.

**Discussion:**

This study will help to clarify whether WBV training improves body balance, gait mobility and muscle strength in frail older persons living in nursing homes. As far as we know, this will be the first study to evaluate the efficacy of WBV for the prevention of falls.

**Trial Registration:**

ClinicalTrials.gov: NCT01375790

## Background

Progressive ageing of the population has generated an increase in chronic diseases [1]. The concomitant increases in morbidity create vulnerability and frailty in older persons.

Frailty is a common syndrome in older persons [2]. Signs and symptoms of this problem are believed to factors such as fatigue, weight loss, exhaustion, weakness, slow walking speed, decreased balance, low levels of physical activity, slowed motor processing and performance, social withdrawal, cognitive changes, and increased vulnerability to stressors [3-6].

Most falls in older persons are caused by frailty [7,8] and multiple medications [9-11]. Research from the United Kingdom [12], the United States [13] and Australia [14] has shown that falls are a tremendous burden to social and health services. Besides affecting psychological and physical health, fractures from falls often imply hospitalization, thereby increasing morbidity. It has been estimated that total health costs attributable to fall-related injuries will practically triple in the next 50 years. Strategies to prevent the negative consequences of falls are needed. One such strategy is exercise [15,16].

Institutionalized older people have less capacity to exercise, and greater osteoarticular deterioration and fatigue than non-institutionalized older people [3]. Improving their physical activity could increase their autonomy and help prevent falls [17-23]. Fighting sedentary attitudes and promoting physical exercise are growing challenges for physiotherapists and other health professionals working in nursing homes.

Whole body vibration is a type of physical exercise that consists of performing static and dynamic exercises on a platform. These effects of this type of exercise have been studied previously in two studies in nursing home residents [24,25]. The results of these interventions were not conclusive because there are differences in the study protocols and limitations in the study designs. Both studies analysed functional capacity and muscle performance but neither recorded the number of falls.

The aim of the present study is to assess the effect of whole-body vibration training on body balance comparing to exercise without vibration in institutionalized older persons. Secondarily, we will evaluate the effects of training on muscular strength and prevention of falls in this population.

## Methods/design

### Study design

**Figure 1:**
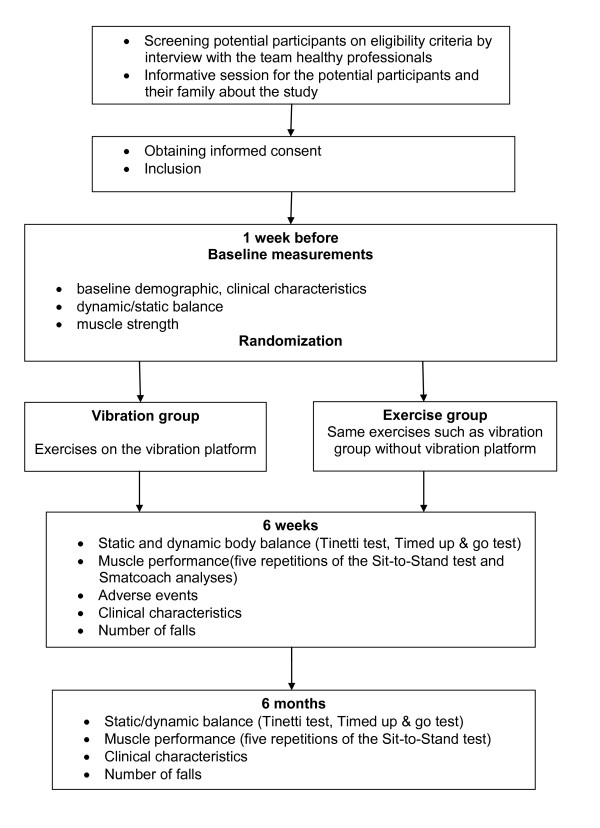
**Flow diagram of the study design**.

This study will be an open randomized, multicentre, and parallel clinical trial with evaluator-blinded. The details of this protocol are reported with CONSORT Statement [26]. Figure 1 presents the flow diagram of the study design. The study protocol was approved by the Ethic Committees of the *Corporació Sanitària Parc Taulí de Sabadell *and, the *Hospital Universitari Mútua de Terrassa*, Spain.

### Participants

One-hundred and sixty volunteers' residents at ten nursing homes in Spain will be included over a period of eleven months (see Additional file 1: List of participating centres).

Clinicians at each nursing home will identify people eligible for the study according to the inclusion and exclusion criteria. Eligible people will be persons of both sexes of seventy-five years or older and living in a nursing home. Inclusions/exclusion criteria are shown in Table 1.

**Table 1:** Inclusion and exclusion criteria

**Inclusion criteria**	65 years and older institutionalized in a nursing home don't have physical and cognitive disorders that they cannot stand in a vibration platform would like to participate in the study
**Exclusion criteria**	acute disease (not resolved during 10 days) pacemaker epilepsy high risk of tromboembolism knee or hip prosthesis musculoskeletal disorders and cognitive or physical dysfunction interfering with test and training procedures

All participants will be informed about the intention of the study and the potential risks before signing the informed consent.

### Interventions

Participants will be randomized to an experimental group (WBV group) or to a control group (exercise group). Participants in the WBV group will perform static/dynamic exercise (balance and resistance training) on a vibratory platform (Frequency: 30-35 Hz; Amplitude: 2-4 mm; Pro5 Airdaptive Model, Power Plate^®^, The Netherlands). Participants in the exercise group will performed the same static/dynamic exercises (balance and resistance training) as the WBV group but without the vibratory platform. Training will consist of 3 sessions per week over 6 weeks. Training volume will be increased progressively over this period. To prevent learning problems at the beginning of the training period, participants will receive an introductory practice session to become familiar with positioning on the vibration platform before the first session [27].

The physiotherapists from each nursing-home will be previously instructed about the study. Three external physiotherapists will verify therapeutic compliance once a week during the intervention and will provide any support needed.

### Follow-up

All tests will be performed at baseline, at 6 weeks (at the end of the intervention

period), and 6 months after the end of the study (Figure 1).

Data concerning falls will be regularly collected from each nursing-home or from relatives if a participant moves to another address.

### Outcome measures

The outcome assessment will be blinded. The two physiotherapists trained in the evaluation procedure will not know the allocation to intervention.

#### Primary outcome

##### • Body Balance

Body balance outcome will be assessed using the Tinetti test [28] and the Timed Up & Go test (TUG) [29].

The Tinetti test has 16 items: 9 for body balance and 7 for gait. The score for each exercise ranges from 0 to 1 or 0 to 2. A lower score indicates poorer physical ability. The global score is 28: 16 is the maximum score for body balance and 12 is the maximum one for gait.

The Timed Up & Go test also assesses functional mobility. Participants stand up from a chair, walk 3 m as quickly and safely as possible, cross a marked line on the floor, turn around and then walk back and sit down in the chair [29]. The chair will be adjusted to the height of each person and will be fixed to the wall. The time taken to complete the task will be recorded.

#### Secondary outcomes

##### • Muscle Performance

Muscle performance will be evaluated using five repetitions of the Sit-to-Stand test [30,31]. Subjects will be asked to stand up and sit down five times, as quickly as possible, with their arms folded across the chest. The chair will be adjusted to the height of each person and will be fixed to the wall. The time taken to complete the task will be recorded by chronometer. The maximum speed of each repetition and the average speed will be recorded using the Smart Coach encoder.

##### • Number of falls

Any falls during the study will be recorded in a fall register developed specifically for the study.

### Sample size

We calculated sample size was calculated assuming a difference of 5 points (SD 10) between the groups in the Tinetti test at the end of intervention. We considered a two-sided alpha level of 0.05, a statistical power of 80%, and 20% of losses [24,32]. The total number of estimated cases was 160 (80 in each group).

### Randomization

A computer generated randomization list will be generated for participants at each nursing-home using the statistical software SPSS17. Allocation to treatment will be centralized by telephone. All the researchers will be blinded to the randomization sequence list.

### Statistical methods

Efficacy will be analysed on an intention-to-treat (ITT) basis. Additionally, a 'per protocol' analysis will be performed. Missing values will be assigned the last available valid score. We will evaluate the effects of the intervention on each quantitative, qualitative and ordinal outcome using the "t" test, Mann-Witney test or the Chi-square test, depending on the type of outcome. The final analysis will be performed 6 weeks and 6 months after randomization. The number of falls over the 6-month period will be recorded.

Changes in body balance and muscle performance will be analysed 6 weeks and 6 months after starting the study using a two-way analysis of variance (ANOVA). Factors included will be the group (vibration or exercise group) and the time (at baseline and after six weeks of intervention), and the interaction between them. The software used will be SPSS17.

## Discussion

WBV training is a type of physical exercise in which people perform various exercises in a squat position on a platform device. Several studies have shown promising results using this intervention in walking capacity, speed of gait, body balance, and muscle strength in elder adults [33-40]. Only two trials have evaluated the feasibility of WBV in functional mobility and muscle performance in institutionalized older persons [24,25] to date. Both these trials were small and the variability of the study protocols, comparators, outcome measures and limitations in the studies design made it difficult to reach any definitive conclusions about the effectiveness of WBV in increasing functional capacity of older persons [41].

Our study design incorporates a significantly larger sample, implements randomization, allocation and blinded outcome assessment, and introduces a longer follow up than previous studies. We consider this design will reveal differences between the two study groups in balance and muscle strength, and secondarily, in preventing falls.

## Competing interests

The authors declare that they have no competing interests. The Tecnosport Condition SLU Company (Badalona, Spain) gives the Power plate vibration platforms for this study (Model Pro5 Airdaptive, Power Plate^®^). Authors have not received remuneration from Tecnosport Condition SLU Company. The clinical trial will be published independently of the positive or negative results.

## Authors' contributions

MSR and MJMZ develop the design of the randomized clinical trial. DRR and AFV provide the intervention support. FRA contributes to the methods of muscular strength assessment. XBC provide the methodological support. All authors have read and approved the final manuscript.

## Pre-publication history

The pre-publication history for this paper can be accessed here:

http://www.biomedcentral.com/1471-2318/11/89/prepub

## Supplementary Material

Additional file 1 **List of Spanish participants centres**. Click here for file
