# Supplementary material for: Whole body vibration for older persons: an open randomized, multicentre, parallel, clinical trial
Source: BMC Geriatr. 2011 Dec 22;11:89. doi: 10.1186/1471-2318-11-89 (PMC3305533; doi:10.1186/1471-2318-11-89)
Supplement: Additional file 1 — List of Spanish participants centres. [file 1471-2318-11-89-S1.DOC]

| 1. Residència Mapfre Quavitae, Barcelona 2. Centre sóciosanitari Albada, Sabadell 3. Residència AMMA Horta, Barcelona 4. Residència Poble Nou, Fundació Vallparadís, Mútua de Terrassa, Barcelona 5. Residència Collserola Mutuam, Barcelona 6. Residència Ballesol Fabra i Puig, Barcelona 7. Residència Ballesol Barberà, Barberà del Vallès 8. Residència Ballesol Badalona, Badalona 9. Residència Ballesol Almogàvers, Barcelona 10. Residència Allegra, Sabadell |
| --- |
